# Niobium oxyhydroxide as a bioactive agent and reinforcement to a high-viscosity bulk-fill resin composite

**DOI:** 10.1590/1678-7757-2023-0278

**Published:** 2024-02-26

**Authors:** Alyssa Teixeira OBEID, Tatiana Rita de Lima NASCIMENTO, Ana Carolina AGASSI, Ana Zélia Falcão ALMEIDA, Ana Paula de Melo Alves GUEDES, João Marco ALVES, Juliana Fraga Soares BOMBONATTI, Marilia Mattar de Amoêdo Campos VELO

**Affiliations:** 1 Universidade de São Paulo Faculdade de Odontologia de Bauru Departamento de Dentística, Endodontia e Materiais odontológicos Bauru Brasil Universidade de São Paulo, Faculdade de Odontologia de Bauru, Departamento de Dentística, Endodontia e Materiais odontológicos, Bauru, Brasil.; 2 Universidade Federal da Paraíba Departamento de Química Centro de Pesquisa de Combustíveis e Materiais João Pessoa Brasil Universidade Federal da Paraíba, Cidade Universitária, Departamento de Química, Centro de Pesquisa de Combustíveis e Materiais (NPE-LACOM), João Pessoa, Brasil.

**Keywords:** Niobium, Composite resins, Dental caries, Nanotechnology, Nanoparticles

## Abstract

**Objective:**

The present *in vitro* study incorporated niobium oxyhydroxide fillers into an experimental high-viscosity bulk-fill resin composite to improve its mechanical performance and provide it a bioactive potential.

**Methodology:**

Scanning electron microscopy synthesized and characterized 0.5% niobium oxyhydroxide fillers, demonstrating a homogeneous morphology that represented a reinforcement for the feature. Fillers were weighed, gradually added to the experimental resin composite, and homogenized for one minute, forming three groups: BF (experimental high-viscosity bulk-fill resin composite; control), BF0.5 (experimental high-viscosity bulk-fill resin composite modified with 0.5% niobium oxyhydroxide fillers), and BFC (commercial bulk-fill resin composite Beautifil Bulk U, Shofu; positive control). In total, 10 specimens/groups (8 × 2 × 2 mm) underwent flexural strength (FS) tests in a universal testing machine (Instron) (500N). Resin composites were also assessed for Knoop hardness (KH), depth of cure (DoC), degree of conversion (DC), elastic modulus (E), and degree of color change (ΔE). The bioactive potential of the developed resin composite was evaluated after immersing the specimens into a simulated body fluid in vitro solution and assessing them using a Fourier-transformed infrared spectroscope with an attenuated total reflectance accessory. One-way ANOVA, followed by the Tukey’s test (p<0.05), determined FS, DC, KH, and ΔE. For DoC, ANOVA was performed, which demonstrated no significant difference between groups (p<0.05).

**Conclusions:**

The high-viscosity bulk-fill resin composite with 0.5% niobium oxyhydroxide fillers showed promising outcomes as reinforcement agents and performed well for bioactive potential, although less predictable than the commercial resin composite with Giomer technology.

## Introduction

The development of bioactive resin composites has gained significant attention over the last decades for controlling tissue loss from dental caries and reducing the risk of secondary caries, one of the primary causes of restoration replacement.^1,2^ Bioactivity refers to the potential of a material to induce apatite mineral nucleation, improving the maintenance of the tooth/material interface^3^ and potentially increasing the clinical longevity of restorations. However, the primary challenge has involved developing materials with adequate remineralization by releasing therapeutic ions or anticaries agents with satisfactory mechanical properties.^4^

Bulk-fill resin composites represent a promising restorative dentistry technique for posterior teeth^5^ and are popular due to the possibility of inserting higher material proportions (4 to 5 mm) than the incremental method with conventional resin composites (up to 2 mm), reducing volume shrinkage.^6^ Their use as a restorative material regards a simplified technique that decreases clinical time, minimizing occasional operator errors and potentially improving patients’ quality of life.^5^

Despite the advantages associated with bulk-fill resin composites,^5^ applying high-viscosity bulk-fill resins to high stress-bearing areas frequently exposed to masticatory forces is still controversial because of their inferior mechanical properties compared to conventional nanohybrid resin composites.^7,8^ A restorative material with minor flexural properties is less capable of initiating and propagating cracks that may fracture within the body and marginal restoration areas,^9,10^ making it more prone to forming gaps and developing caries adjacent to rehabilitations (secondary caries).^9^ Therefore, a high-viscosity bulk-fill resin composite with intrinsic bioactive ability may improve the mechanical properties and affect the long-term performance of restorative materials.

Adding different nanostructures to resin composites has improved their mechanical properties.^11,12,13^ Overall, the type or concentration of these structures affects mechanical features,^14^ such as degree of conversion (DC) and microhardness (KH), considering that the refractive index of nanostructures may decrease light energy availability within the polymer.^15^ Studies have demonstrated that high-viscosity bulk-fill resin composites present lower DC and depth of cure (DoC) than conventional ones.^16,17^ Thus, incorporating a nanostructure that increases the mechanical strength of high-viscosity bulk-fill resin composites without impairing DC and DoC would be interesting to improve clinical performance with the bulk-fill technique, expanding clinical applications.

Niobium oxides present remarkable physicochemical properties with high mechanical stability in many hostile environments.^18^ Despite the limitations of using niobium oxide as a reinforcement for dental materials, several studies have demonstrated its bioactive potential in dental composites due to its bioactivity and ability to grow hydroxyapatite crystals in contact with human saliva.^19,20^ Furthermore, using niobium oxide as a filler for dental materials may reduce costs and availability limitations during material development since Brazil has the largest niobium reserves in the world.^18^

Conversely, the lack of chemical interactions on the surface of oxides may cause weak bonding interactions within organic matrices with unstable chemical bonds and no interlocking strength. To overcome that, oxide surface functionalization enables nanostructures to remain chemically stable, preventing agglomeration due to the increased nanostructure dispersion throughout the resin composite matrix.^21^ Niobium oxyhydroxide (or niobium acid) is responsible for high chemical stability and numerous active sites, and its functionalization and incorporation into resin composites may improve the performance of materials working as reinforcement and bioactive fillers. Moreover, niobium oxyhydroxide presents high catalytic activity and may form electron pairs and increase material polymerization when exposed to light irradiation.^22^

Therefore, this study evaluated the influence of incorporating niobium oxyhydroxide fillers into an experimental high-viscosity bulk-fill resin composite, analyzing their Knoop hardness (KH), depth of cure (DoC), degree of conversion (DC), elastic modulus (E), and degree of color change (∆*E*). It also assessed the bioactive potential of the experimental high-viscosity bulk-fill resin composite customized with niobium oxyhydroxide fillers using a Fourier-transformed infrared (FTIR) spectroscope with an attenuated total reflectance (ATR) accessory.

## Methodology

### Experimental design

**Figure 1:**
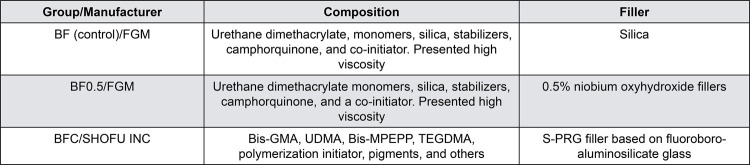
Chemical composition of the experimental resin-based composites

The experimental bulk-fill resin composites were produced by FGM Produtos Odontológicos LTDA (Joinville, Santa Catarina, Brazil) and prepared with a resin matrix formulated with urethane dimethacrylate monomers, silica, stabilizers, camphorquinone, and a co-initiator, presenting high viscosity. They were divided into three groups: BF/control: experimental high-viscosity bulk-fill resin composite; BF0.5: experimental high-viscosity bulk-fill resin composite with 0.5% niobium oxyhydroxide fillers; and BFC/positive control: commercial bulk-fill resin composite (Beautifil Bulk U, Shofu). Figure 1 describes the composition, fillers, and manufacturers of materials characterized by KH, DoC, DC, E, ∆*E*, and bioactivity.

SigmaPlot 12.0 (Systat Software, San Jose, CA, USA) determined sample size. This study used a 0.05 significance level and 80% power.

### Synthesis of niobium oxyhydroxide fillers and scanning electron microscopy (SEM)

Silanized niobium was achieved in two steps: i) synthesis and ii) silanization. First, the acid was synthesized under a process that consisted of preparing a 0.26mol L^-1^ solution of the precursor salt - niobium ammonium oxalate - and then gradually adding 1 mol L^-1^ of a sodium hydroxide solution under constant agitation at 65°C. Alkaline solution addition stopped when the mixture reached a pH of 7, remaining in a 70-°C oven for 72 hours. The precipitate was washed several times with distilled water. Finally, the solid was dried in a 70-°C oven for 24 hours, and its granulometry was standardized in a 200 mesh.

**Figure 2:**
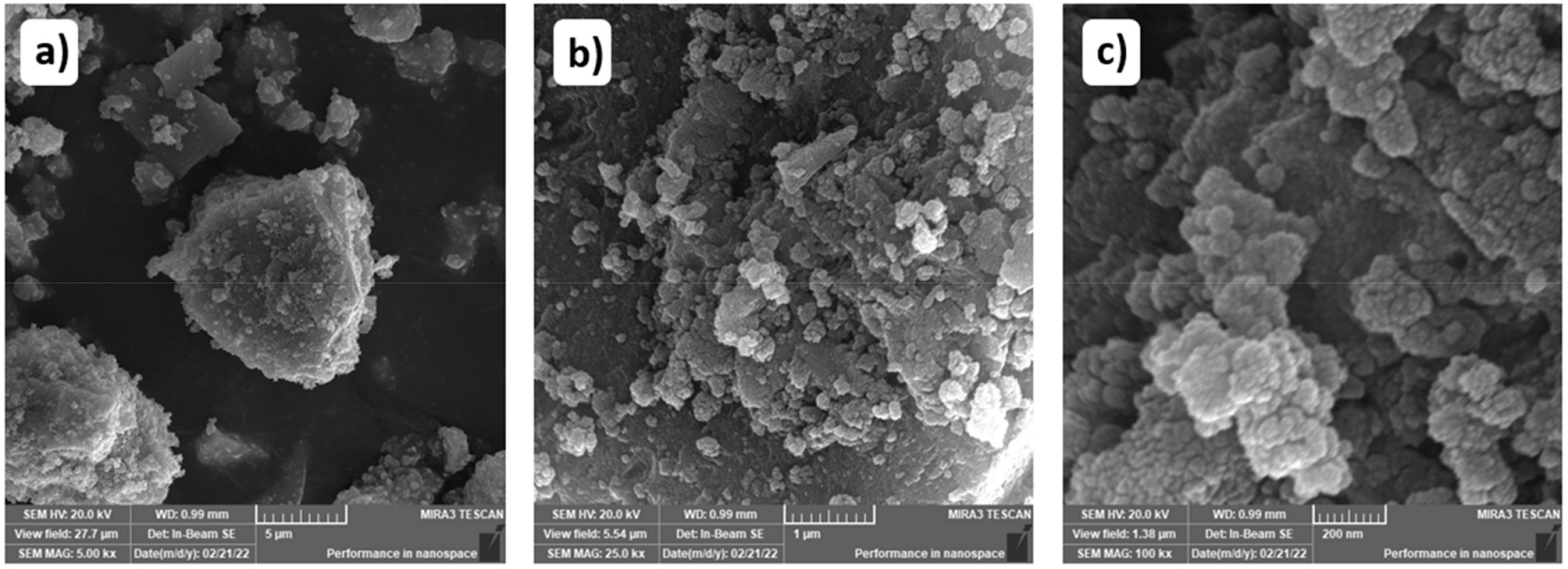
FEG-SEM images of niobium oxyhydroxide with dimensions of a) 5,000; b) 25,000; and c) 100,000x

Secondly, the nanoparticles of niobium were silanized with the (3-mercaptopropyl) trimethoxysilane (MPTMS) silylating agent by a silanization reaction based on the methodology by Queiroga, et al.^23^ (2019) applied to bentonites. The acid was previously dried in a 100-°C oven for 24 hours. Then, the solid was dispersed in xylene, followed by 10 mL of silane, which were added under constant mechanical agitation for 48 hours in a 100-°C nitrogen atmosphere. Next, the solid was washed with xylene and then ethanol and dried in a 70-°C oven for 24 hours. A Tescan microscope, MIRA 3 model, with magnifications of 5,000, 25,000, and 100,000× provided the scanning electron microscopy (SEM) analysis of niobium oxyhydroxide, as in Figure 2.

**Figure 3:**
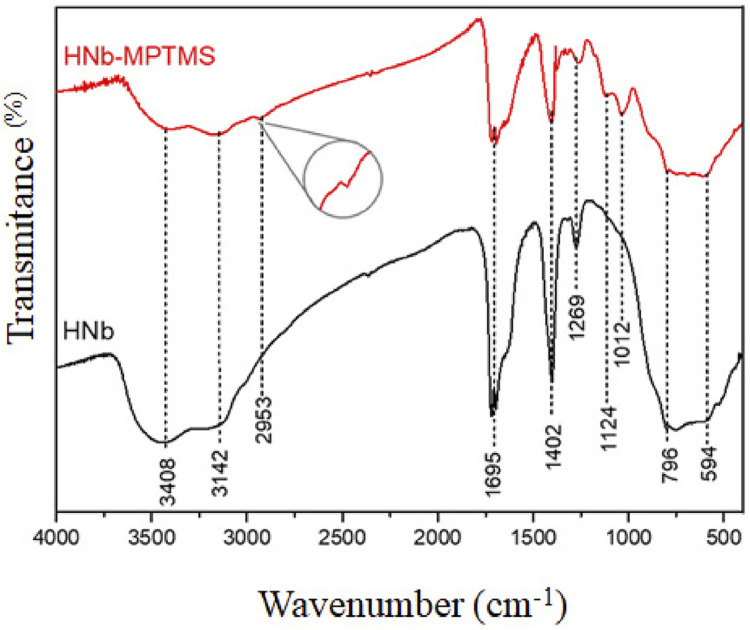
FTIR spectra of HNb and HNb-MPTMS

**Figure 4:**
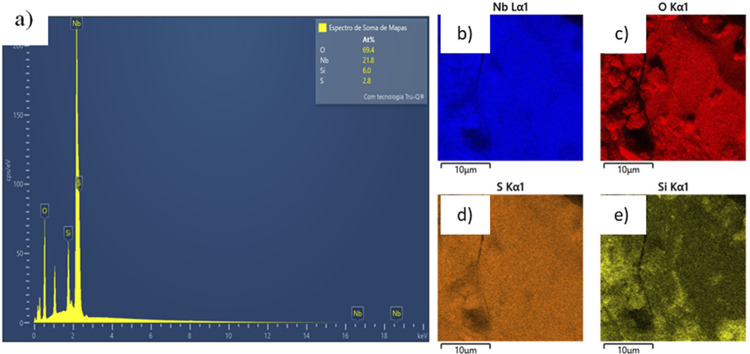
EDS spectrum (a) and EDS maps for niobium (b), oxygen (c), sulphur (d), and silicon (e) for the solid HNb-MPTMS

Fourier-transform infrared absorption spectroscopy (FTIR) was performed using a Shimadzu spectrophotometer, IR Prestige-21 model, in transmission mode (Figure 3). KBr pellets were used in the 4000-400 cm^-1^ range, with a resolution of 4 cm^-1^ and 20 accumulations. High resolution images of the solids and qualitative silanization dispersion (composition) of the samples were characterized by Field Emission Scanning Electron Microscopy (FEG-SEM) and Energy Dispersive X-ray Spectroscopy (EDS) using Tescan brand equipment – model FEG Mira 3 – LMH (Figure 4).

### Development of an experimental high-viscosity bulk-fill resin

All experimental groups used a resin composite incorporated with nanoparticles. A 0.0001-g precision scale (Denver Instrument, São Paulo, Brazil) weighed each material dose, calculating the nanostructure values corresponding to the percentage by resin composite weight. The niobium oxyhydroxide fillers were weighed, added to the resin gradually, and homogenized for one minute.

### Knoop microhardness (KH) and depth of cure (DoC)

Disc-shaped samples (4 × 4 mm^2^) (n=6) were prepared by placing the material on stainless steel molds covered with a polyester strip. The sample surfaces were polished using decreasing-grit abrasive papers (600, 800, and 1200, Buehler Ltd., Lake Bluff, IL, USA) for two minutes each, followed by a 0.5mm diamond paste (Buehler Ltd., Lake Bluff, IL, USA). The top and bottom of each specimen received three indentations along a midline with 100-mμ spacings (Knoop diamond 50 g, dwell-time 10 seconds) using digital microhardness equipment (Micromet II, Buehler, USA). The mean of the three readings was obtained for each sample. The bottom/top percent ratio of KH values was calculated to determine DoC.^24^

### Degree of conversion (DC)

FTIR-ATR (IR Prestige-21 Shimadzu; Tokyo, Japan) (n=5/group) evaluated DC with a 4-cm^1^ resolution and 32 scans from 4000 to 800 cm^1^. All specimens were analyzed after mixing the resin composite with the nanoparticles. They were immediately placed over the ATR crystal to obtain the baseline spectra. DC calculations used the intensities of the aliphatic C = C absorption peak at 1638 cm^1^ to the carbonyl group C = O peak at 1608 cm^1^ between polymerized and uncured resin composites.^25^ The degree of conversion percentage (%DC) was calculated for each specimen as follows:


$$\text{ DC }\%=\frac{[1-\text{ peak height after curing }]\times 100}{\text{ Peak height before curing }}$$

### Flexural strength (FS) and elastic modulus (E)

Bar-shaped samples measuring 8 × 2 × 2 mm (n=10) were prepared by pouring the described mixtures into stainless-steel split molds and modifying the sample length to prevent overexposed or uncured regions considering the diameter of the LED-curing device, pre-calibrated with 1.000 mW/cm^2^ for 40 s (VALO; Ultradent, Utah, USA).^20^

The tests were conducted in a universal testing machine (Instron 5943, Norwood, MA, USA) equipped with a 500-N load cell at a crosshead speed of 0.5 mm/min according to ASTM D 790-86. Samples were loaded in three-point bending using a 6-mm span length on the top surface of each specimen. The following equation determined the values:


$$FS=\frac{3FL}{2bd2}$$

Where *FS* is the flexural strength in Mpa, *F* is the loading force at the fracture point, *L* is the support span length (6 mm), and *b* and *d* are the width and thickness, respectively.

#### Degree of color change (∆E)

The ∆E test (n=7) assessed color changes at different time points using a CIELab-based colorimeter (Vita Easyshade V; Vita Zahnfabrik). The spectrophotometer was calibrated before the measurements according to the manufacturer’s instructions. An initial measurement (P0) was taken 24 hours after specimen production; a second one (P1), seven days after P0; and a third measurement, after artificial aging (P2), consisting of a 24-hour water storage at 60ºC.^26^ All specimens were dry-stored at 37ºC without light between P0 and P1. In total, three consecutive measurements were made in the center of each specimen until value uniformity was obtained.

∆E calculations used the following equation: $\Delta E=\sqrt{}\left({DL}^{*}\right)2+\left({Da}^{*}\right)+\left({Db}^{*}\right)2$ , where DL*, Da*, and Db* correspond to the color differences between baseline (P0) and after the storage period (P1 and P2).

## Bioactivity analysis

The glass slide used to develop the samples for this test was 1-mm thick, and the disc-shaped samples (4 × 4 mm^2^) were prepared by placing the material on a stainless steel mold covered with a polyester strip over the glass slide, creating the 5-mm light tip-to-material polymerization distance.^27^ Then, the Valo light tip was carefully centered on the sample and light-cured with a LED-curing device, measured as previously described (1.000 mW/cm^2^ for 40 seconds; VALO; Ultradent, Utah, USA).^20,28^

The solution was prepared with the following reagents: sodium chloride (NaCl), potassium chloride (KCL), di-potassium hydrogen phosphate trihydrate (K_2_HPO_4_.3H_2_O), magnesium chloride hexahydrate (MgCl_2_.6H_2_O), calcium chloride (CaCl_2_), sodium sulfate (Na_2_SO_4_), Tris-hydroxymethyl aminomethane (Tris) buffer, and 1M hydrochloric acid. The reagents were immersed in purified water and maintained in a magnetic stirrer. The pH was verified and adjusted to 7.4.

Overall, two specimens from each group (BF, BF0.5, and BFC) (n=3) were immersed in 5 mL of the described solution and maintained in airtight containers in a 37-**°C** oven for the determined times: T0 - initial time, T1 - the first hour after immersing the specimens in the solution, T14 - 14 days after specimen immersion, and T21 - 21 days after specimen immersion.

The resin composites were subjected to Fourier-transform infrared spectroscopy (FTIR - Shimadzu Corporation, Model IR Prestige 21, Kyoto, Japan) analysis at the initial time (zero), after one hour, and after 14 and 21 days of immersion in the solution.

## Statistical analysis

The SigmaPlot software, version 12.0 (Systat Software, San Jose, CA, USA), was used to statistically analyze the data. The Shapiro-Wilk test verified normal distribution and equality of variances for all variables. One-way ANOVA, followed by the Tukey’s test (p<0.05), determined FS, DC, and KH. The Shapiro-Wilk test analyzed E data as they showed abnormality (p<0.05). For DoC, ANOVA was performed, which demonstrated no significant difference between groups (p<0.05). Bioactivity data were qualitatively collected, thus dispensing with statistical analyses.

The Q-Q Plot with the simulated envelope verified the assumption of normality of ∆E value residuals. The parametric analysis data were compared with non-parametric test findings provided by ATS statistics (ANOVA Type-Statistic).

## Results

### SEM, FTIR, and mechanical properties

SEM images (Figure 2) showed irregular particle clusters without a second phase. The FTIR spectra presented in Figure 3 showed the characteristic bands of niobium oxyhydroxide. These bands initially appear at 3408 and 3142 cm^-1^, corresponding to the O–H stretching in the Nb–OH bond on the surface and in the bulk, respectively.^29^ Meanwhile, the region from 796 to 594 cm^-1^ corresponds to the vibrations of the Nb–O–Nb bonds. According to Oliveira, et al.^30^ (2015), these bands are crucial for identifying the bonds involving niobium and oxygen. Additionally, images showed spectral bands at 1695 cm^-1^ associated with surface-adsorbed water, as well as regions at 1402 and 1269 cm^-1^, which are related to impurities in the niobium precursor salt.^31^

Table 1 demonstrates that DC was similar between BF0.5 and BFC (p>0.05) without statistically significant differences. That indicates that the monomer conversion of the bulk-fill resin composite with 0.5% niobium oxyhydroxide fillers (BF0.5) was similar to the commercial resin composite (BFC). However, the experimental resin composite used as control (BF) showed a significantly higher DC (p<0.05) than the other two groups. Although the DC of BF0.5 was lower than BF, the values were similar to BFC, and the DoC of the three groups was also comparable (p>0.05) (Table 2). The control (BF) and experimental (BF0.5) groups showed higher FS than the commercial resin (BC) (p<0.001) (Table 1). As for KH values, group BF0.5 was superior to BF and BFC for top and bottom surfaces (p=0.001) (Table 2).

**Table 1:** Values of the degree of monomer conversion (%, n=5), flexural strength (mean ± standard deviation, n=10), and elastic modulus (median-interquartile range, n=10) of the tested groups

Groups	Degree of conversion (%)	Flexural strength (FS, Mpa) (mean±SD)	Elastic modulus (E, GPa) (median-IQR)
BF	68.8±1.6^b^	100.8±13.6^a^	6.5(6.3-6.7)^a^
BF0.5	36.2±2.1^a^	104±17^a^	6.5(6.3-6.8)^a^
BFC	38±3.5^a^	74±12^b^	6.0(5.6-6.6)^a^

Values in the same column with different superscript lowercase letters significantly differ from each other (p<0.05).

**Table 2:** Top surface hardness (mean ± standard deviation, n=6), bottom surface hardness (mean ± standard deviation, n=6), and calculated depth of cure (%, median-interquartile range) of the tested groups

Groups	Knoop hardness (top) (Kg/mm^**2**^)	Knoop hardness (bottom) (Kg/mm^**2**^)	Depth of cure (%DoC) (median-IQR)
BF	63±3.9^a^	47.3±3.2	75.2(6.5-2.3)^a^
BF0.5	70±5.6^b^	55±5	78.3(5.8-2)^a^
BFC	55.2±2.4^c^	43±1.4	78.1(4.9-1.7)^a^

Values in the same column with different superscript lowercase letters significantly differ from each other (p<0.05).

**Figure 5:**
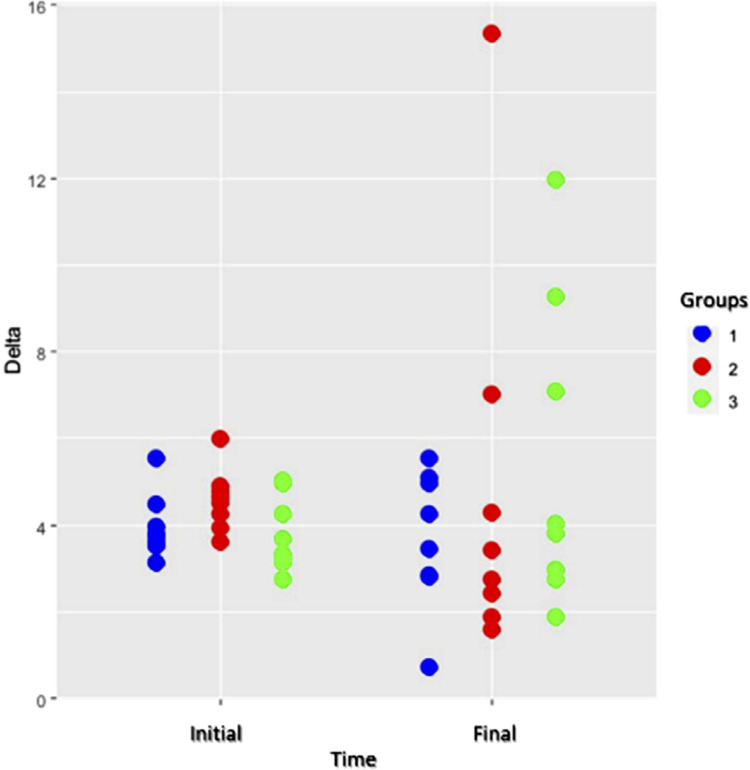
Chart of individual values for the degree of color change (ΔE).

∆E at the initial time showed similar behavior in all three groups. Delta variability increased at the final time and was more evident in group 3. However, the groups showed no statistical differences (p=0.92) (Figure 5).

### Bioactivity

**Figure 6:**
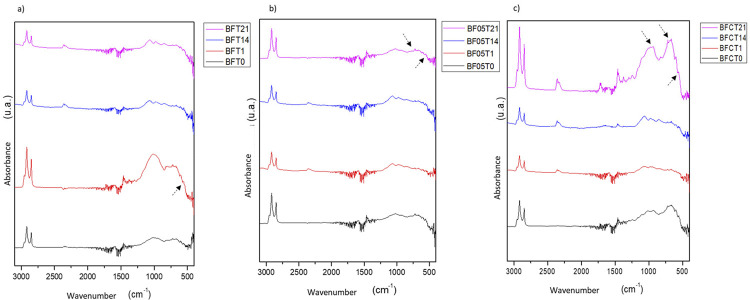
(a) FTIR spectra in the samples of BF referring to the studied times (T0, T1, T14, and T21).(b) FTIR spectra in the samples of BF0.5 referring to the studied times (T0, T1, T14, and T21). (c) FTIR spectra in the samples of BFC referring to the studied times (T0, T1, T14, and T21)

Figure 6 represents the spectra of samples for all tested materials: (a) BF (control), (b) BF0.5 (resin composite doped with 0.5% of niobium), and (c) BFC (commercial resin composite with Giomer technology) in all evaluated times (T0, T1, T14, and T21). FTIR analysis showed free phosphate (PO^4-^) bands captured by the surface area of the resin composites in all groups at the peaks of ~560 within T1 (BF) and T21 (BF0.5 and BFC). After 1 h of immersion in the solution, P-O bands suggested a rapid deposition of calcium phosphate at the surface area of the samples (Figure 6a, arrow).

Figure 6b (arrow) showed a deposition of PO^4-^ on the surface of the doped resin composite after 21 days of immersion. During this period, peaks of ~560 and ~600 appeared, typical of apatite formation. These bands may have peak replacement due to the presence of other C-O bands also found in the control group (BF).

The FTIR spectra at 21 days in Figure 6c showed peaks at ~560, ~600, and ~950 and a typical halo of phosphate bands (PO^4-^) at ~950-1050 (arrows), which may indicate depositions of apatite layers on the surface of the material, showing its greater bioactivity capacity.

## Discussion

This study used 0.5% niobium oxyhydroxide fillers to synthesize the experimental resin composite since the fraction of bioactive fillers in the material should remain minimal to promote remineralization by ion release without affecting mechanical properties.^32^For the functionalized niobium oxyhydroxide material (HNb-MPTMS), the FTIR spectrum profile was very similar. However, this study found a distinct band was observed, serving as an indicator of the presence of silane in the 2953 cm^-1^ region. This observation is attributed to the vibrations of the C-H bonds in the organic compound.^33^ Another evidence supporting the anchoring of MPTMS in the niobium structure was confirmed by the absorption band at 1124 cm^-1^, corresponding to Si-C bond vibrations,^34^ and at 1012 cm^-1^, associated with siloxanes (Si-O).^23^

Based on the micrographs (Figure 2), a homogeneous morphology with clustered particles was observed. Silanization was once again confirmed by the presence of silicon and sulfur in the EDS data presented in Figure 4.

Over the years, hybrid resin composites have been developed as bioactive restorative materials to expand the clinical indications of bulk-fill resin composites. They interfered with caries development adjacent to restorations due to their therapeutic function of releasing ions when in contact with the oral environment.^35^ This technology, known as Giomer, combines the advantages of glass ionomers (anticariogenic and self-adhesive properties) while addressing their poor esthetics and possible dehydration issues by a pre-reacted glass-ionomer filler surface incorporated into resin composites, offering esthetics and high bond strength.^12^

Although studies have found inferior physicomechanical properties for hybrid resin composites compared to conventional and high-viscosity bulk-fill nanocomposites,^11,13,36^ this study found a similar DC between groups BF0.5 and BFC (p>0.05) (Table 1) without statistically significant differences. A correlation between filler components, such as size, distribution, and flexural properties, should be considered, indicating that Giomer resin composites have higher filler contents^16^ but increased bioactivity potential even after a long period. Another aspect to consider is that DC may highly depend on the quality of the three-dimensional polymeric network formed after polymerization and the variation in filler quantity. The relationship between DC and other mechanical properties is not always straightforward. DC decreases proportionally with increasing filler contents, probably due to light scattering at resin-filler interfaces.^37^Moreover, incorporating fillers may increase the viscosity of some dental materials, possibly explaining their lower DC.^38^ However, the values in group BF0.5 neither affected their mechanical properties nor statistically differed from the commercial resin (group BFC).

DoC was similar in all groups (p>0.05), potentially due to the clinical translucency similarity of the tested resin composites (BF and BFC) enabling effective polymerization inside the polymer (Table 2). Although filler incorporation can alter refractive index and light dispersion, the catalytic activity of niobium in BF0.5 might contribute to this effective polymerization rate.^22^

Although the flexural strength data in BF0.5 failed to significantly differ from the other groups, they achieved a minimum of 80 MPa for polymer-based restorative materials, according to ISO 4049:2019. Flexural strength was the primary indicator of the physicomechanical properties of the bulk-fill resin composite in this study because it creates tensile, compressive, and shear stresses, representing a satisfactory parameter to provide meaningful insights into the fracture strength of a material. Thus, testing under the most challenging mechanical conditions may reduce the chances of accepting a material that fails prematurely due to inadequate strength.^39^ Moreover, the survival rate and wear resistance of Giomer resin composites met ADA guidelines for tooth-colored restorative materials in posterior teeth even after four years.^36,40^ Therefore, comparing a low concentration of niobium fillers in a high-viscosity bulk-fill resin composite with Giomer technology might be a satisfactory parameter to measure its performance in this study.

The color stability of a restorative material determines restoration success and patients’ acceptance. Optical properties usually improve when nanoparticles are incorporated into restorative materials because nanoparticles are smaller than the wavelength of visible light, demonstrating better light transmittance.^41^ The data in this study suggest that all tested materials had similar color stability even after aging (including the control group), helping to extend the lifespan of restorative materials.

Fourier-transform infrared spectroscopy analysis assessed bioactivity by comparing different release times. Bioactive materials have a biological effect or are biologically active, bonding tissues and materials^42^ according to their potential to induce specific and intentionally desired mineral attachment to the dentin substrate.^43^ This study used simulated body fluid (SBF) to mimic biomimetic mineralization, providing suitable temperature, ion concentration, and pH conditions similar to human blood plasma. SBF offers an adequate supersaturated environment around the substrate and facilitates bone-like apatite depositions.^44^ PO^4-^ deposition occurred on the surface of samples containing niobium. Characteristic apatite formations appeared after 21 days, but an EDS analysis could complement these results. The resin composite that showed the greatest peak intensity refers to BFC at 21 days.

However, many clinicians still suspect that the mechanical properties of bulk-fill materials might be unsuitable for clinical use in posterior teeth,^16^ preferring to avoid using the 4-5mm increments recommended by manufacturers. Conversely, providing a bioactive potential to bulk-fill resin composites can be promising and attractive because these materials are used in deep cavities with larger increments than conventional composites. Further studies evaluating the use of increments up to 5 mm should be performed to find the most effective thickness for high-viscosity bulk-fill resin composites with niobium nanoparticles.

## Conclusion

The high-viscosity bulk-fill resin composite with 0.5% niobium oxyhydroxide fillers presented promising outcomes as a reinforcement agent, showing good bioactive potential despite its lower predictability than the commercial resin composite with Giomer technology.
